# Prognostic Factors and Survival in Non-Small Cell Lung Cancer Patients Treated with Chemoradiotherapy

**DOI:** 10.3889/oamjms.2015.003

**Published:** 2014-12-29

**Authors:** Simonida Crvenkova

**Affiliations:** University Clinic of Radiotherapy and Oncology, Faculty of Medicine, Ss Cyril and Methodius University of Skopje, Skopje, Republic of Macedonia

**Keywords:** prognostic factors, performance status, stage, weight loss, conformal radiotherapy, sequential and concurrent chemoradiotherapy

## Abstract

**BACKGROUND:**

According to the literature, performance status, stage-tumor dimension and nodal status, weight loss, were the most important prognostic factors for survival in patients with locally advanced non-small cell lung cancer.

**AIM:**

To evaluate the treatment results and the prognostic variables in our patients treated with sequential and concurrent chemoradiotherapy.

**MATERIAL AND METHODS:**

In the study 85 patients were randomly assigned to one of the two treatment arms. In the sequential arm, 45 patients had previously received sequential chemotherapy with 4 cycles of and etoposide followed by conformal radiotherapy (RT). In the second concurrent group, 40 patients received concomitant chemotherapy of cisplatine and etoposide and conformal RT, followed by two cycles of consolidation chemotherapy of carboplatine and etoposide. We described all phases of the conformal three dimensional (3-D) RT.

**RESULTS:**

From October 2005 to March 2008, 93 patients were enrolled. Eight patients were not eligible, seven had stage IV and one patient had pleural effusion. They were all initially considered to have stage IIIB disease. The median survival was 13 months for the patients in the sequential arm and 19 months for those in the concurrent treatment arm. The differences were statistically significant (log-rank test p=0.0039). The disease-free survival was 9 months in the sequential arm and 16 months in the concurrent treatment group. The differences were statistically significant (log-rank test p=0.0023). We found that the following prognostic factors significantly influenced the survival in lung cancer patients treated with conservative method: - age, p<0.05; - performant status, p<0.001; - weight loss, p<0.001; tumor dimension, p<0.05; and - nodal involvement, p<0.05.

**CONCLUSION:**

In our study, the dose-limiting toxicity, esophagitis was reduced by performing conformal radiotherapy. Conformal thoracic radiotherapy and new radiotherapy technics, such as respiratory gated radiotherapy, allow dose escalating and may probably improve survival and local control in lung cancer patients.

## Introduction

Lung cancer remains a worldwide epidemic. Approximately 1.2 million people die from lung cancer each year. NSCLC represents >80% of all lung cancers. 60%-70% of the patients with NSCLC suffer stage III or IV disease. In the late 1980s, radiotherapy was the standard treatment for these patients [1]. Randomized trials and a 1995 overview subsequently showed that combination/combined chemoradio-therapy was superior to radiotherapy alone [2]. Many chemotherapeutic agents active in NSCLC possess radiosensitizing properties, thereby improving the probability of local control. In addition, chemotherapy administered concurrently with thoracic radiation may act systemically and potentially eradicate distant micrometastases. Several studies showed the feasibility of the cisplatin-etoposide combination plus radiotherapy for patients with stage III disease [3].

Successfully tailored therapy in lung cancer patients requires defining the prognostic factors. Prognostic factors are defined as characteristics of patients and stage, before starting the treatment. Analyzing these characteristics provides the opportunity to select patients in different groups and choose the best treatment modality for the selected group. They are also important to evaluate the results of treatments and to compare the results of different clinical studies [4].

Primary end point of this study was to evaluate the treatment results and the prognostic variables in our patients treated with sequential and concurrent chemoradiotherapy.

## Material and Methods

One hundred and ten patients with NSCLC stage IIIA and IIIB were analyzed at the University Clinic of Radiotherapy and Oncology in Skopje in the period from October 2005 to March 2008. Only 85 patients were eligible for this study, aged between 18 and 70 years, had an Eastern Cooperative Oncology Group (ECOG) Score ≤ 1, and had ≤ 10 % weight loss in the period of 3 months before inclusion. They have previously untreated histological or cytological proven NSCLC, unrespectable stage IIIA-N2 disease, or stage IIIB disease without pleural effusion. Stage IIIB disease was assigned either by N3 (contralateral mediastinal or supraclavicular nodes) or by T4 from invasion of mediastinal structures. Following prognostic factors were evaluated: aged (18-43, 44-55, 56-70); histological type (squamous, adenocarcinoma large cell carcinoma and unspecified type); performance status (PS) according to ECOG Score ≤ 1, weight loss in the period of 3 months before inclusion (< 5%, 5-10%); lymph nodes involvement and tumor dimension (≤5 cm, > 5 cm and unmeasurment because atelectasis, pneumonitis); symptoms duration before treatment (< 3 months, 3-6 months, > 6 months); and hemoglobin level (<120 g/l, ≥ 120 g/l).

The following laboratory values were required: leucocytes ≥ 1.5x10³/l, platelets ≥ 100x10/l, AST and ALT ≤ 2 x the upper limit of the reference range. Ineligibility criteria were as follows: uncontrolled infection, or fever over 38ºC, unstable cardiovascular disease and previous malignancy.

Before enrolment, the patients gave their full medical histories and underwent clinical examination with assessment of performance status (PS). In the sequential arm, responses were assessed 8 weeks after the end of radiotherapy. In the concurrent arm, responses were assessed 8 weeks after the end of the consolidation chemotherapy. Imaging studies x-ray and/or computed tomography (CT) could be repeated at all times when clinically indicated. Complete and partial responses were based on RECIST criteria. Toxicity was graded according to RTOG/EORTC criteria. Follow-up visits were conducted every 2 months during the first year and, afterwards, every 3 months. Patients were randomly assigned to receive sequential or concurrent therapy. In the sequential arm, 45 patients received four cycles of chemotherapy. They were administered first, consisting of carboplatin (AUC x 6) on day 1 and etoposide on days 1-3, repeated every 3 weeks. The radiotherapy began 4 weeks after the fourth cycle of chemotherapy administration. Chemotherapy and radiotherapy began simultaneously in concurrent arm, including 40 patients. The radiotherapy schedule was identical to that in the sequential arm. The first cycle with cisplatin 30 mg/m^2^ and etoposide 100mg/m^2^ was administered on days 1 to 3 and the second 3-days cycle was administered the last 3 days of radiotherapy. After 4 weeks of concurrent chemoradiotherapy schedule, two cycles of consolidation chemotherapy began, consisting of carboplatine (AUC x 6) and etoposide 100 mg/m^2^ on day 1 to 3.

**Figure 1:**
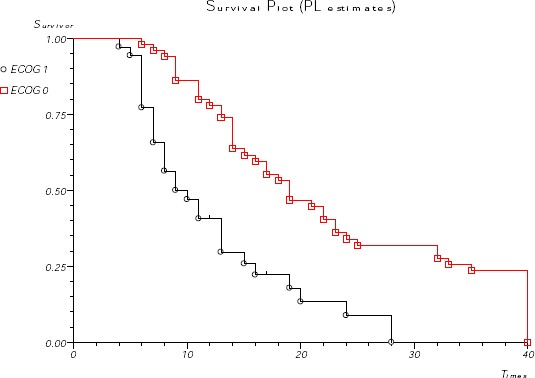
*ECOG status and survival*.

Conformal radiotherapy at both arms consisted of 60 Gy in 30 fractions of 2 Gy per fraction, for 5 days a week given over a period of 6 weeks. Treatment planning CT was required to define the gross tumor volume (GTV). Each patient was positioned in an immobilization device-wing board in the treatment position on a flat table. CT slices with 5 mm thickness were obtained, starting from cricoid cartilage and extending inferiorly to the level of the L1 vertebral body. The GTV, clinical target volume (CTV), planning target volume (PTV) and normal organs were outlined on all CT slices (Figure 1).

**Figure 2:**
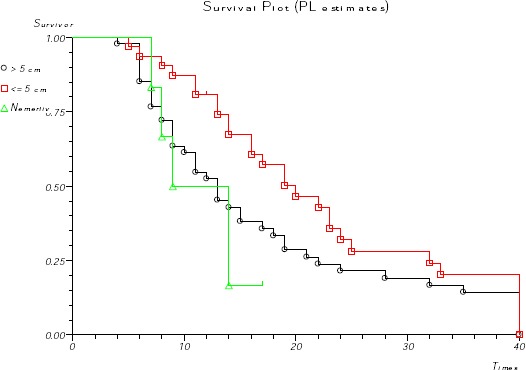
*Survival according to tumor dimension*.

The normal tissues that were contoured included both lungs (as the total lung volume), heart, spinal cord and esophagus. The CTV included the entire GTV plus 0.7 cm and the PTV included CTV plus another 0.7 cm adding margin. PTV44 was treated with parallel-opposed anterior-posterior fields and PTV60 was treated with any combination of fields depends of spinal cord constrain (Figure 2).

If radiotherapy had to be delayed for more than 7 days, the patient was withdrawn from the study. Patients with evidence of progression at any time were removed from the study, but continued to be evaluated for survival and toxicity. Survival and interval to recurrence or progression were measured from the date of the first treatment session.

## Results

From October 2005 to March 2008, 93 patients were enrolled. Eight patients were not eligible, seven had stage IV and one patient had pleural effusion. All these were initially considered to have stage IIIB disease. The characteristics of 85 patients are listed in Table 1.

**Table 1:** The characteristics of 85 patients.

Patient characteristics	Concurrent chemoradiotherapy arm	Sequential chemoradiotherapy arm	p
No of eligible pts	40	45	

Age			
18-43		1	0.13
44-55	20	13	
56-70	20		

Sex			
Male	35	40	0.98
Female	5	5	

Performance status			
0	26	23	0.19
1	14	22	

Weight loss			
<5%	26	23	0.13
5-10%	14	22	

Histology			
Squamous cell	22	34	0.26
Adenocarcinoma	10	6	
Large cell	3	2	
Unspecified	5	3	

N status			
N1	12	15	0.93
N2	25	27	
N3	3	3	

Tumor ≤ 5 sm	14	18	0.38	
Tumor > 5 sm	27	20

Hemoglobin			
<120 g/l	11	19	0.15
≥120 g/l	29	26	

Duration of symptoms			
< 3 months	2	0	0.29
3-6 months	21	23	
> 6 months	17	22	

**Figure 3:**
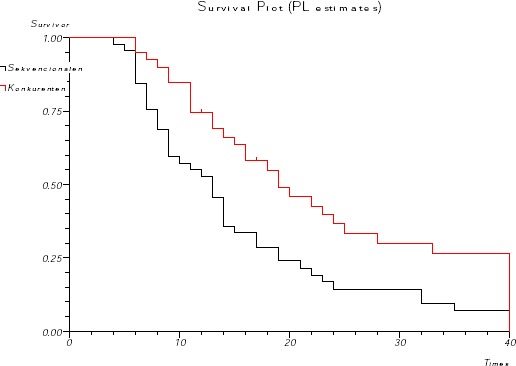
*Overall survival according to the treatment*.

**Figure 4:**
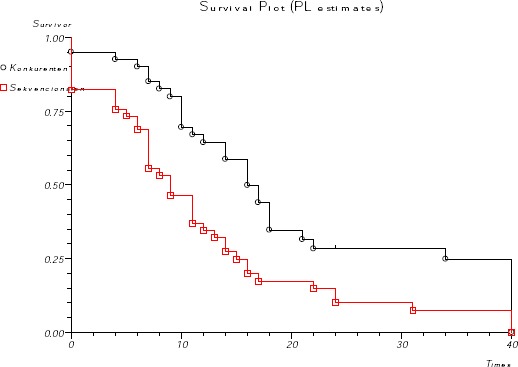
*Disease-free survival according to the treatment*.

Survival was analyzed until July 2009, after a median follow-up of 3 years. The median survival was 13 months in the sequential arm (95%CI 10.2-15.7), and 19 months in the concurrent treatment arm (95%CI 13.6-24.3). The differences were statistically significant (log-rank test p = 0.0039, Figure 3). The 1-year, 2-year and 3-year survival rates were 74%, 36% and 27% in the concurrent arm and 52%, 14% and 7.1% in the sequential arm, respectively. Disease free survival for the concurrent arm was 16 months (95% CI 12.7-19.2), and for the sequential arm was 9 months (95% CI 5.8- 12.16). The differences were statistically significant (log-rank test p = 0.0023 Figure 4).

One of the aims of this study was to evaluate the prognostic factors. The analysis was not performed according to the treatment modality: sequential or concurrent, because the two groups were homogeneous without significant differences between them.

Most patients in our study were between 44-55 and 56-70 years of age. The youngest patient was 38 years old and the oldest was 70 years old. Average age was 58.2 years (SD ± 6.68 years). Patients aged between 44 and 55 had median survival of 14 months (95% CI 10.15-17.84), significantly better than the patients between 56 and 70 years of age, with median survival time of 10 months (95% CI 4.3-15.6) (p<0.05).

As we expected, performance status, according to ECOG criteria, has important influence on survival. Overall survival for patients with ECOG 0 was 19 months, significantly better than 10 months for patients with ECOG 1 status (p<0.001), (Figure 1).

Another prognostic factor influencing the survival was weight loss in the period of 3 months before inclusion. Median survival in patients group with weight loss under 5% was 16 months (95% CI 12.2 months to 19.7 months) and was, in statistical terms, significantly better than 7 months (95% CI 5.5 months to 11.3 months) in patients with weight loss between 5% and 10% (p<0.001).

Tumor dimension was also an important prognostic factor with statistical influence on survival (p<0.001). Patients with tumor dimension under 5 cm in size have median survival of 20 months (95% CI 13.6-26.3), compared with survival of 13 months at patients with tumors dimension over 5 cm in size (95% CI 9.8 months to 16.1 months) and 9 months (95% CI 6.1 months to 13.8 months) at unmeasured tumors (Figure 2).

Lymph nodes involvements also have statistical influence on survival in lung cancer patients treating with chemoradiotherapy. Patients with N1 lymph nodes involvement live the longest median of 20 months (95% CI 11.1 months to 32.8 months), compared to 13 months (95% CI 9.6 months to 16.3 months) at patients with N2 involvements and 7 months (95% CI 5.8 -8.1) at those patients with N3 involvement (p<0.001).

Another parameter we analyzed in this study was hemoglobin level in patients’ blood. Patients with hemoglobin level under 120 g/l had survival average of 9 months (95% CI 6.5 -11.4), and patients with hemoglobin level over 120 g/l had survival of approximately 17 months (95% CI 12.9-21.1). The differences between two groups of patients were high, but still not statistically significant (p=0.06), (Table 2).

**Table 2:** Survival according to hemoglobin level.

Hemoglobin level	Average (months)	12 Months (%)	24 Months (%)	36 Months (%)	Total
<120 g/l	9	44%	14%	0%	30 (35%)

≥120 g/l	17	72%	29%	16%	55 (65%)

		p = 0.06			85 (100%)

In our study histological type of lung tumors, gender and duration of symptoms, before starting the treatment, were prognostics factors without any significant influence on survival.

## Discussion

Our study compared sequential and concurrent chemoradiation therapy in locally advanced NSCLC. We found more benefit of concurrent therapy than in the previously listed trials, in terms of overall and disease-free survival (19 vs 13; 16 vs 9 months), and the difference was significant with log-rank test. When our study was designed, the cisplatin-etoposide combination was mostly used concurrently with radiotherapy. Consolidation chemotherapy with two cycles of carboplatin-etoposide was administered in the concurrent arm to balance the dose of platinum based chemotherapy in the two arms. This consolidation chemotherapy administered after concurrent chemoradiotherapy seems promising in terms of survival, as shown in the Southwest Oncology Group (SWOG) S9504 and Locally Advanced Multimodality Protocol (LAMP) studies [5, 6]. In the SWOG S9504 study, consolidation docetaxel following concurrent chemoradiotherapy has shown median survival of 26 months and median progression free survival of 16 months. Our concurrent-consolidation arm showed similar results (OS 19 months and DFS 16 months). In our study, the local relapse rate was lower in the concurrent arm than in the sequential arm. In the RTOG 94-10 [7] study, local failure rates at 2 years were significantly lower in the concurrent arm. Thus, it seems that the superior survival observed with concurrent treatment is associated with better local control [8-10].

Sealy at all [11] informed that elderly patients with inoperable lung cancer manifested local disease recurrence, while younger patient showed distant dissemination, proven in our study. According to the most authors, gender did not influence survival.

According to the literature, number of female with lung cancer was increasing with decreasing ratio male/female. We did not confirm this in our study, because, in Macedonia, smoking among the female population was rarity [12].

According to our study, histology type of lung cancer did not influence the survival, but same data from the literature confirm that squamous lung cancer patients live longer than those with adenocarcinoma [13].

We have confirmed in our study that tumor dimension was an important factor influencing the survival. Perez informed that that there was only correlation between tumor dimension and local disease control without influence on the survival [14].

The most important prognostic factor according to the literature was the performance status. We also confirmed that the performance status before starting the treatment had statistically significant influence on the survival [15].

In our study, median survival in patients’ group with weight loss under 5% was 16 months and was significantly better, in statistical terms, than 7 months in patients’ group with weight loss between 5 and 10%. Data from the literature confirmed our conclusion [16].

We highly recommend precisely defining the stage of disease and the prognostic factors in lung cancer patients and hoping that in better selected patient group the chance for giving best treatment will be higher.

Given the high toxicity in the concurrent-consolidation schedule, it should be reserved for patients younger than 70 years, having good performance status and minimal weight loss. In our study, the dose-limiting toxicity, esophagitis was reduced by performing conformal radiotherapy. Conformal thoracic radiotherapy and new radiotherapy techniques, such as respiratory gated radiotherapy, allow dose escalating and may probably improve survival and local control in lung cancer patients.
